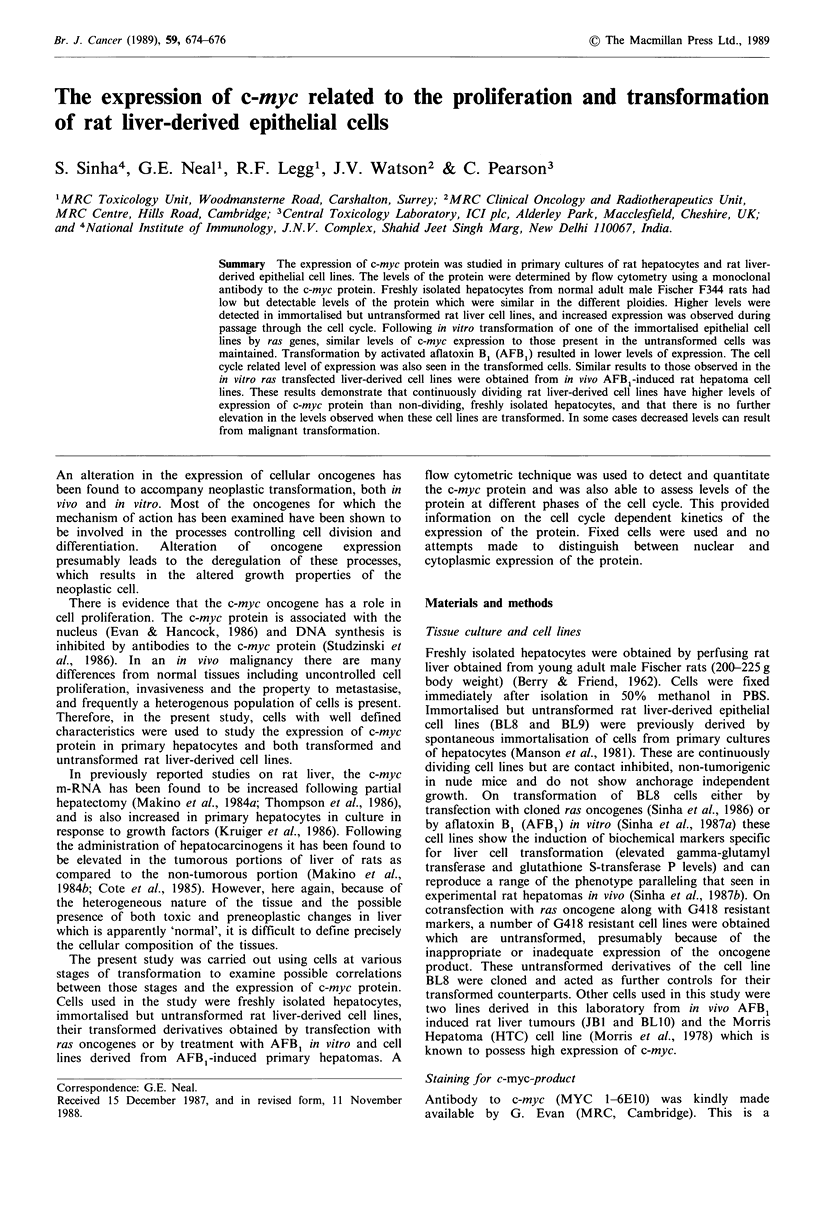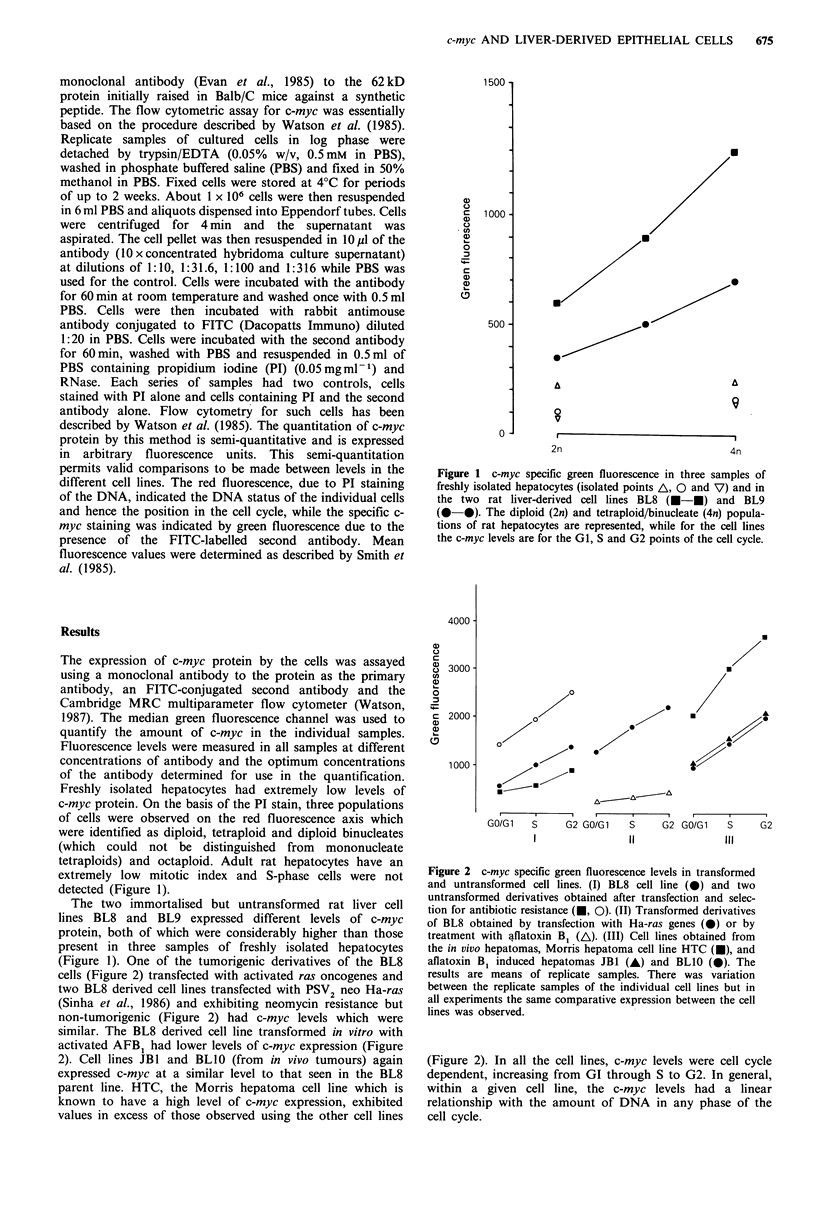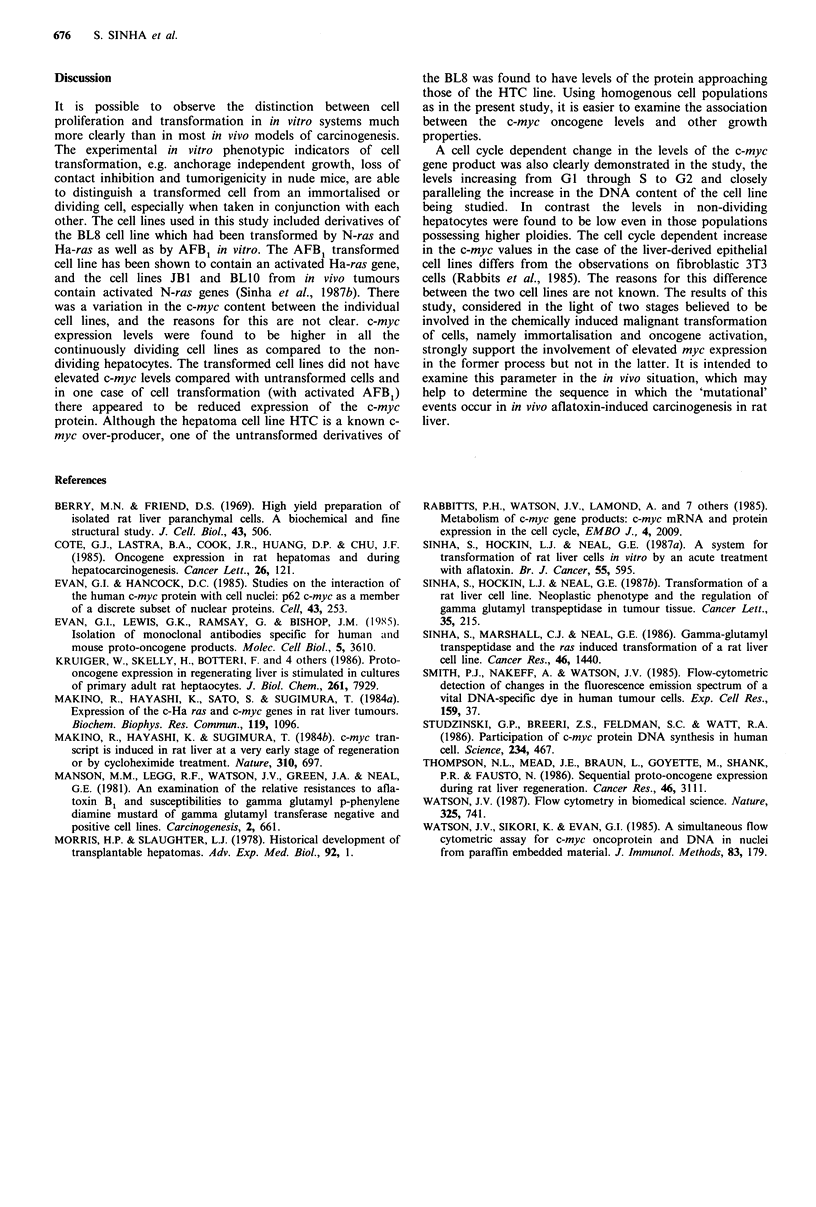# The expression of c-myc related to the proliferation and transformation of rat liver-derived epithelial cells.

**DOI:** 10.1038/bjc.1989.139

**Published:** 1989-05

**Authors:** S. Sinha, G. E. Neal, R. F. Legg, J. V. Watson, C. Pearson

**Affiliations:** National Institute of Immunology, J.N.V. Complex, Shahid Jeet Singh Marg, New Delhi, India.

## Abstract

The expression of c-myc protein was studied in primary cultures of rat hepatocytes and rat liver-derived epithelial cell lines. The levels of the protein were determined by flow cytometry using a monoclonal antibody to the c-myc protein. Freshly isolated hepatocytes from normal adult male Fischer F344 rats had low but detectable levels of the protein which were similar in the different ploidies. Higher levels were detected in immortalised but untransformed rat liver cell lines, and increased expression was observed during passage through the cell cycle. Following in vitro transformation of one of the immortalised epithelial cell lines by ras genes, similar levels of c-myc expression to those present in the untransformed cells was maintained. Transformation by activated aflatoxin B1 (AFB1) resulted in lower levels of expression. The cell cycle related level of expression was also seen in the transformed cells. Similar results to those observed in the in vitro ras transfected liver-derived cell lines were obtained from in vivo AFB1-induced rat hepatoma cell lines. These results demonstrate that continuously dividing rat liver-derived cell lines have higher levels of expression of c-myc protein than non-dividing, freshly isolated hepatocytes, and that there is no further elevation in the levels observed when these cell lines are transformed. In some cases decreased levels can result from malignant transformation.


### The expression of c-*myc* related to the proliferation and transformation of rat liver-derived epithelial cells

S. Sinha[4], G.E. Neal[1], R.F. Legg[1], J.V. Watson[2] & C. Pearson[3]

[1]*MRC Toxicology Unit, Woodmansterne Road, Carshalton, Surrey;* [2]*MRC Clinical Oncology and Radiotherapeutics Unit, MRC Centre, Hills Road, Cambridge;* [3]*Central Toxicology Laboratory, ICI plc, Alderley Park, Macclesfield, Cheshire, UK; and* [4]*National Institute of Immunology, J.N.V. Complex, Shahid Jeet Singh Marg, New Delhi 110067, India.*

**Summary** The expression of c-*myc* protein was studied in primary cultures of rat hepatocytes and rat liver-derived epithelial cell lines. The levels of the protein were determined by flow cytometry using a monoclonal antibody to the c-*myc* protein. Freshly isolated hepatocytes from normal adult male Fischer F344 rats had low but detectable levels of the protein which were similar in the different ploidies. Higher levels were detected in immortalised but untransformed rat liver cell lines, and increased expression was observed during passage through the cell cycle. Following *in vitro* transformation of one of the immortalised epithelial cell lines by *ras* genes, similar levels of c-*myc* expression to those present in the untransformed cells was maintained. Transformation by activated aflatoxin $B_1$ ($AFB_1$) resulted in lower levels of expression. The cell cycle related level of expression was also seen in the transformed cells. Similar results to those observed in the *in vitro ras* transfected liver-derived cell lines were obtained from *in vivo* $AFB_1$-induced rat hepatoma cell lines. These results demonstrate that continuously dividing rat liver-derived cell lines have higher levels of expression of c-*myc* protein than non-dividing, freshly isolated hepatocytes, and that there is no further elevation in the levels observed when these cell lines are transformed. In some cases decreased levels can result from malignant transformation.

An alteration in the expression of cellular oncogenes has been found to accompany neoplastic transformation, both *in vivo* and *in vitro*. Most of the oncogenes for which the mechanism of action has been examined have been shown to be involved in the processes controlling cell division and differentiation. Alteration of oncogene expression presumably leads to the deregulation of these processes, which results in the altered growth properties of the neoplastic cell.

There is evidence that the c-*myc* oncogene has a role in cell proliferation. The c-*myc* protein is associated with the nucleus (Evan & Hancock, 1986) and DNA synthesis is inhibited by antibodies to the c-*myc* protein (Studzinski *et al.*, 1986). In an *in vivo* malignancy there are many differences from normal tissues including uncontrolled cell proliferation, invasiveness and the property to metastasise, and frequently a heterogenous population of cells is present. Therefore, in the present study, cells with well defined characteristics were used to study the expression of c-*myc* protein in primary hepatocytes and both transformed and untransformed rat liver-derived cell lines.

In previously reported studies on rat liver, the c-*myc* m-RNA has been found to be increased following partial hepatectomy (Makino *et al.*, 1984*a*; Thompson *et al.*, 1986), and is also increased in primary hepatocytes in culture in response to growth factors (Kruiger *et al.*, 1986). Following the administration of hepatocarcinogens it has been found to be elevated in the tumorous portions of liver of rats as compared to the non-tumorous portion (Makino *et al.*, 1984*b*; Cote *et al.*, 1985). However, here again, because of the heterogeneous nature of the tissue and the possible presence of both toxic and preneoplastic changes in liver which is apparently 'normal', it is difficult to define precisely the cellular composition of the tissues.

The present study was carried out using cells at various stages of transformation to examine possible correlations between those stages and the expression of c-*myc* protein. Cells used in the study were freshly isolated hepatocytes, immortalised but untransformed rat liver-derived cell lines, their transformed derivatives obtained by transfection with *ras* oncogenes or by treatment with $AFB_1$ *in vitro* and cell lines derived from $AFB_1$-induced primary hepatomas. A flow cytometric technique was used to detect and quantitate the c-*myc* protein and was also able to assess levels of the protein at different phases of the cell cycle. This provided information on the cell cycle dependent kinetics of the expression of the protein. Fixed cells were used and no attempts made to distinguish between nuclear and cytoplasmic expression of the protein.

Correspondence: G.E. Neal.
Received 15 December 1987, and in revised form, 11 November 1988.

### Materials and methods

### *Tissue culture and cell lines*

Freshly isolated hepatocytes were obtained by perfusing rat liver obtained from young adult male Fischer rats (200–225 g body weight) (Berry & Friend, 1962). Cells were fixed immediately after isolation in 50% methanol in PBS. Immortalised but untransformed rat liver-derived epithelial cell lines (BL8 and BL9) were previously derived by spontaneous immortalisation of cells from primary cultures of hepatocytes (Manson *et al.*, 1981). These are continuously dividing cell lines but are contact inhibited, non-tumorigenic in nude mice and do not show anchorage independent growth. On transformation of BL8 cells either by transfection with cloned *ras* oncogenes (Sinha *et al.*, 1986) or by aflatoxin $B_1$ ($AFB_1$) *in vitro* (Sinha *et al.*, 1987*a*) these cell lines show the induction of biochemical markers specific for liver cell transformation (elevated gamma-glutamyl transferase and glutathione S-transferase P levels) and can reproduce a range of the phenotype paralleling that seen in experimental rat hepatomas *in vivo* (Sinha *et al.*, 1987*b*). On cotransfection with *ras* oncogene along with G418 resistant markers, a number of G418 resistant cell lines were obtained which are untransformed, presumably because of the inappropriate or inadequate expression of the oncogene product. These untransformed derivatives of the cell line BL8 were cloned and acted as further controls for their transformed counterparts. Other cells used in this study were two lines derived in this laboratory from *in vivo* $AFB_1$ induced rat liver tumours (JB1 and BL10) and the Morris Hepatoma (HTC) cell line (Morris *et al.*, 1978) which is known to possess high expression of c-*myc*.

### *Staining for* c-myc-*product*

Antibody to c-*myc* (MYC 1–6E10) was kindly made available by G. Evan (MRC, Cambridge). This is a monoclonal antibody (Evan *et al.*, 1985) to the 62 kD protein initially raised in Balb/C mice against a synthetic peptide. The flow cytometric assay for c-*myc* was essentially based on the procedure described by Watson *et al.* (1985). Replicate samples of cultured cells in log phase were detached by trypsin/EDTA (0.05% w/v, 0.5 mM in PBS), washed in phosphate buffered saline (PBS) and fixed in 50% methanol in PBS. Fixed cells were stored at 4°C for periods of up to 2 weeks. About $1 \times 10^6$ cells were then resuspended in 6 ml PBS and aliquots dispensed into Eppendorf tubes. Cells were centrifuged for 4 min and the supernatant was aspirated. The cell pellet was then resuspended in 10 $\mu$l of the antibody (10 x concentrated hybridoma culture supernatant) at dilutions of 1:10, 1:31.6, 1:100 and 1:316 while PBS was used for the control. Cells were incubated with the antibody for 60 min at room temperature and washed once with 0.5 ml PBS. Cells were then incubated with rabbit antimouse antibody conjugated to FITC (Dacopatts Immuno) diluted 1:20 in PBS. Cells were incubated with the second antibody for 60 min, washed with PBS and resuspended in 0.5 ml of PBS containing propidium iodine (PI) (0.05 mg ml$^{-1}$) and RNase. Each series of samples had two controls, cells stained with PI alone and cells containing PI and the second antibody alone. Flow cytometry for such cells has been described by Watson *et al.* (1985). The quantitation of c-*myc* protein by this method is semi-quantitative and is expressed in arbitrary fluorescence units. This semi-quantitation permits valid comparisons to be made between levels in the different cell lines. The red fluorescence, due to PI staining of the DNA, indicated the DNA status of the individual cells and hence the position in the cell cycle, while the specific c-*myc* staining was indicated by green fluorescence due to the presence of the FITC-labelled second antibody. Mean fluorescence values were determined as described by Smith *et al.* (1985).

### Results

The expression of c-*myc* protein by the cells was assayed using a monoclonal antibody to the protein as the primary antibody, an FITC-conjugated second antibody and the Cambridge MRC multiparameter flow cytometer (Watson, 1987). The median green fluorescence channel was used to quantify the amount of c-*myc* in the individual samples. Fluorescence levels were measured in all samples at different concentrations of antibody and the optimum concentrations of the antibody determined for use in the quantification. Freshly isolated hepatocytes had extremely low levels of c-*myc* protein. On the basis of the PI stain, three populations of cells were observed on the red fluorescence axis which were identified as diploid, tetraploid and diploid binucleates (which could not be distinguished from mononucleate tetraploids) and octaploid. Adult rat hepatocytes have an extremely low mitotic index and S-phase cells were not detected (Figure 1).

The two immortalised but untransformed rat liver cell lines BL8 and BL9 expressed different levels of c-*myc* protein, both of which were considerably higher than those present in three samples of freshly isolated hepatocytes (Figure 1). One of the tumorigenic derivatives of the BL8 cells (Figure 2) transfected with activated *ras* oncogenes and two BL8 derived cell lines transfected with $PSV_2$ neo Ha-*ras* (Sinha *et al.*, 1986) and exhibiting neomycin resistance but non-tumorigenic (Figure 2) had c-*myc* levels which were similar. The BL8 derived cell line transformed *in vitro* with activated $AFB_1$ had lower levels of c-*myc* expression (Figure 2). Cell lines JB1 and BL10 (from *in vivo* tumours) again expressed c-*myc* at a similar level to that seen in the BL8 parent line. HTC, the Morris hepatoma cell line which is known to have a high level of c-*myc* expression, exhibited values in excess of those observed using the other cell lines

**Figure 1** c-*myc* specific green fluorescence in three samples of freshly isolated hepatocytes (isolated points △, ○ and ▽) and in the two rat liver-derived cell lines BL8 (■—■) and BL9 (●—●). The diploid (2*n*) and tetraploid/binucleate (4*n*) populations of rat hepatocytes are represented, while for the cell lines the c-*myc* levels are for the G1, S and G2 points of the cell cycle.

**Figure 2** c-*myc* specific green fluorescence levels in transformed and untransformed cell lines. (I) BL8 cell line (●) and two untransformed derivatives obtained after transfection and selection for antibiotic resistance (■, ○). (II) Transformed derivatives of BL8 obtained by transfection with Ha-*ras* genes (●) or by treatment with aflatoxin $B_1$ (△). (III) Cell lines obtained from the *in vivo* hepatomas, Morris hepatoma cell line HTC (■), and aflatoxin $B_1$ induced hepatomas JB1 (▲) and BL10 (●). The results are means of replicate samples. There was variation between the replicate samples of the individual cell lines but in all experiments the same comparative expression between the cell lines was observed.

(Figure 2). In all the cell lines, c-*myc* levels were cell cycle dependent, increasing from GI through S to G2. In general, within a given cell line, the c-*myc* levels had a linear relationship with the amount of DNA in any phase of the cell cycle.

### Discussion

It is possible to observe the distinction between cell proliferation and transformation in *in vitro* systems much more clearly than in most *in vivo* models of carcinogenesis. The experimental *in vitro* phenotypic indicators of cell transformation, e.g. anchorage independent growth, loss of contact inhibition and tumorigenicity in nude mice, are able to distinguish a transformed cell from an immortalised or dividing cell, especially when taken in conjunction with each other. The cell lines used in this study included derivatives of the BL8 cell line which had been transformed by N-*ras* and Ha-*ras* as well as by $AFB_1$ *in vitro*. The $AFB_1$ transformed cell line has been shown to contain an activated Ha-*ras* gene, and the cell lines JB1 and BL10 from *in vivo* tumours contain activated N-*ras* genes (Sinha *et al.*, 1987*b*). There was a variation in the c-*myc* content between the individual cell lines, and the reasons for this are not clear. c-*myc* expression levels were found to be higher in all the continuously dividing cell lines as compared to the non-dividing hepatocytes. The transformed cell lines did not have elevated c-*myc* levels compared with untransformed cells and in one case of cell transformation (with activated $AFB_1$) there appeared to be reduced expression of the c-*myc* protein. Although the hepatoma cell line HTC is a known c-*myc* over-producer, one of the untransformed derivatives of the BL8 was found to have levels of the protein approaching those of the HTC line. Using homogenous cell populations as in the present study, it is easier to examine the association between the c-*myc* oncogene levels and other growth properties.

A cell cycle dependent change in the levels of the c-*myc* gene product was also clearly demonstrated in the study, the levels increasing from G1 through S to G2 and closely paralleling the increase in the DNA content of the cell line being studied. In contrast the levels in non-dividing hepatocytes were found to be low even in those populations possessing higher ploidies. The cell cycle dependent increase in the c-*myc* values in the case of the liver-derived epithelial cell lines differs from the observations on fibroblastic 3T3 cells (Rabbits *et al.*, 1985). The reasons for this difference between the two cell lines are not known. The results of this study, considered in the light of two stages believed to be involved in the chemically induced malignant transformation of cells, namely immortalisation and oncogene activation, strongly support the involvement of elevated *myc* expression in the former process but not in the latter. It is intended to examine this parameter in the *in vivo* situation, which may help to determine the sequence in which the 'mutational' events occur in *in vivo* aflatoxin-induced carcinogenesis in rat liver.

### References

BERRY, M.N. & FRIEND, D.S. (1969). High yield preparation of isolated rat liver paranchymal cells. A biochemical and fine structural study. *J. Cell. Biol.*, **43**, 506.

COTE, G.J., LASTRA, B.A., COOK, J.R., HUANG, D.P. & CHU, J.F. (1985). Oncogene expression in rat hepatomas and during hepatocarcinogenesis. *Cancer Lett.*, **26**, 121.

EVAN, G.I. & HANCOCK, D.C. (1985). Studies on the interaction of the human c-*myc* protein with cell nuclei: p62 c-*myc* as a member of a discrete subset of nuclear proteins. *Cell*, **43**, 253.

EVAN, G.I., LEWIS, G.K., RAMSAY, G. & BISHOP, J.M. (1985). Isolation of monoclonal antibodies specific for human and mouse proto-oncogene products. *Molec. Cell Biol.*, **5**, 3610.

KRUIGER, W., SKELLY, H., BOTTERI, F. and 4 others (1986). Proto-oncogene expression in regenerating liver is stimulated in cultures of primary adult rat heptaocytes. *J. Biol. Chem.*, **261**, 7929.

MAKINO, R., HAYASHI, K., SATO, S. & SUGIMURA, T. (1984*a*). Expression of the c-Ha *ras* and c-*myc* genes in rat liver tumours. *Biochem. Biophys. Res. Commun.*, **119**, 1096.

MAKINO, R., HAYASHI, K. & SUGIMURA, T. (1984*b*). c-*myc* transcript is induced in rat liver at a very early stage of regeneration or by cycloheximide treatment. *Nature*, **310**, 697.

MANSON, M.M., LEGG, R.F., WATSON, J.V., GREEN, J.A. & NEAL, G.E. (1981). An examination of the relative resistances to aflatoxin $B_1$ and susceptibilities to gamma glutamyl p-phenylene diamine mustard of gamma glutamyl transferase negative and positive cell lines. *Carcinogenesis*, **2**, 661.

MORRIS, H.P. & SLAUGHTER, L.J. (1978). Historical development of transplantable hepatomas. *Adv. Exp. Med. Biol.*, **92**, 1.

RABBITTS, P.H., WATSON, J.V., LAMOND, A. and 7 others (1985). Metabolism of c-*myc* gene products: c-*myc* mRNA and protein expression in the cell cycle, *EMBO J.*, **4**, 2009.

SINHA, S., HOCKIN, L.J. & NEAL, G.E. (1987*a*). A system for transformation of rat liver cells *in vitro* by an acute treatment with aflatoxin. *Br. J. Cancer*, **55**, 595.

SINHA, S., HOCKIN, L.J. & NEAL, G.E. (1987*b*). Transformation of a rat liver cell line. Neoplastic phenotype and the regulation of gamma glutamyl transpeptidase in tumour tissue. *Cancer Lett.*, **35**, 215.

SINHA, S., MARSHALL, C.J. & NEAL, G.E. (1986). Gamma-glutamyl transpeptidase and the *ras* induced transformation of a rat liver cell line. *Cancer Res.*, **46**, 1440.

SMITH, P.J., NAKEFF, A. & WATSON, J.V. (1985). Flow-cytometric detection of changes in the fluorescence emission spectrum of a vital DNA-specific dye in human tumour cells. *Exp. Cell Res.*, **159**, 37.

STUDZINSKI, G.P., BREERI, Z.S., FELDMAN, S.C. & WATT, R.A. (1986). Participation of c-*myc* protein DNA synthesis in human cell. *Science*, **234**, 467.

THOMPSON, N.L., MEAD, J.E., BRAUN, L., GOYETTE, M., SHANK, P.R. & FAUSTO, N. (1986). Sequential proto-oncogene expression during rat liver regeneration. *Cancer Res.*, **46**, 3111.

WATSON, J.V. (1987). Flow cytometry in biomedical science. *Nature*, **325**, 741.

WATSON, J.V., SIKORI, K. & EVAN, G.I. (1985). A simultaneous flow cytometric assay for c-*myc* oncoprotein and DNA in nuclei from paraffin embedded material. *J. Immunol. Methods*, **83**, 179.